# Sperm Sexing in Selected Animals and Humans: Methods, Applications, and Future Prospects

**DOI:** 10.1111/andr.70179

**Published:** 2026-01-29

**Authors:** Domrazek Kinga, Jurka Piotr

**Affiliations:** ^1^ Institute of Veterinary Medicine Warsaw University of Life Sciences Warsaw Poland

**Keywords:** flow cytometry, Percoll, semen, sperm sexing, swim‐up

## Abstract

**Background:**

Sperm sexing is a technique that enables the selection of offspring sex by sorting spermatozoa based on their sex chromosomes. This technology has gained increasing attention due to its potential applications in both animal breeding and human‐assisted reproduction.

**Applications:**

In livestock production, sperm sexing offers substantial economic and genetic benefits, including increased milk production in dairy cattle, improved meat yields in beef cattle, and the prevention of sex‐linked genetic diseases. In human reproduction, sex selection techniques may support family balancing and reduce the risk of transmitting hereditary disorders. Commonly used methods, such as flow cytometry and density gradient centrifugation, have been progressively refined to enhance sorting efficiency and accuracy.

**Challenges:**

Despite its advantages, sperm sexing presents technical limitations and raises ethical concerns, particularly regarding its societal implications and the welfare of embryos selected through assisted reproductive technologies.

**Conclusions and Future Perspectives:**

This review summarizes current sperm sexing methods and their applications in animals and humans, highlights existing challenges, and discusses future directions for technological advancement. The development of safer, more effective, and ethically acceptable approaches may further expand the role of sperm sexing in sustainable animal production and personalized reproductive medicine.

## 1 Introduction

Sperm sexing is a reproductive technology that sorts live spermatozoa by their sex chromosomes. This technique enables the production of offspring with a predetermined sex, which is particularly valuable for managing sex‐linked genetic conditions [1].

One of the primary motivations is economic efficiency. It allows animal farmers to select the desired sex for economic advantages—such as increasing milk production by obtaining more female calves in dairy farming, while beef producers might favor male calves for their faster growth rates [2]. Additionally, using sexed semen can aid in producing genetically superior animals and help maintain balanced sex ratios within a herd, thereby optimizing overall breeding strategies.

In humans, the main determinants for choosing sex selection are following: having sons or daughters according to cultural norms, fulfilling specific economic roles within a family, creating symmetric siblings sex compositions and establishing chosen birth order sequences. New reproductive technologies have facilitated these practices and have also been used to prevent births affected by specific sex‐linked genetic disorders [3]. From this perspective, human sperm sexing is a promising technical alternative because it may reduce reliance on embryo selection; however, it also raises ethical concerns—including risks of reinforcing gender bias, potential sex‐ratio distortions, and non‐medical use—so it is not inherently more ethical than embryo selection [4].

This review critically examines the currently available sperm sexing methods across selected animal species and humans, highlighting both the biological principles and reported outcomes. Special attention is given to the efficacy, limitations, and practical applications of each technique, as well as emerging technologies and species‐specific challenges.

### 1.1 Historical Context

Obtaining the desired sex of offspring has been the subject of debate since ancient times. Democritus (470–402 BC) proposed that the right testis was responsible for producing male offspring, while the left testis produced female offspring [5]. The ideas of various ancient thinkers, such as Hippocrates and Aristotle, regarding the factors determining sex were pre‐scientific theories of reproduction. When the father of Western medicine, Hippocrates, first speculated that child sex might be determined by physical conditions such as body temperature and other factors, he was distancing himself from folklore‐based arguments [6]. Aristotle and others later popularized variations of these beliefs, including the notion that the side of the uterus were semen was deposited would determine a baby's sex. He taught that higher temperatures led to the conception of male offspring with stronger, more potent semen, while lower temperatures would produce female subjects [7]. These philosophical theories prevailed until the Antonie van Leeuwenhoek used a microscope to observe spermatozoa for the first time, describing them as “animalcules” [8]. In 1905, while researching the mealworm beetle (*Tenebrio molitor*), Nettie Maria Stevens discovered that male beetles had a chromosome noticeably smaller than those found in females. She identified this smaller chromosome as the “Y” chromosome, which, along together with the larger “X” chromosome, plays a key role in determining the sex of the organism. Her work established the fundamental understanding of how X and Y chromosomes contribute to sex determination [9]. In the 1980s, Johnson et al. evaluated sperm sexing by using flow cytometry [10, 11]. In 1990s the commercial application of sperm sorting became common in farm animals [12]. Now the methods of sperm sexing applied in veterinary and human medicine [13, 14, 15]. In humans, this technology still arouses controversy owing to ethical aspects [16].

### 1.2 Scope of the Review

This review aims to provide a comparative evaluation of sperm sexing methods (Table 1) across three key species of interest: humans and selected animals. While numerous reviews have addressed sperm sexing in a general context or focused predominantly on livestock [17, 18, 19, 20], this paper emphasizes cross‐species differences and similarities, highlighting methodological performance, limitations, and feasibility in each species. Special attention is given to emerging technologies such as magnetic‐activated cell sorting (MACS), molecular sexing, and genome‐editing tools (e.g., CRISPR/Cas) that may revolutionize future applications.

The review also explores the practical and ethical implications of sperm sexing in both agricultural breeding systems and assisted human reproduction, including its use for disease prevention and family balancing. While applications in conservation biology are important, they fall outside the scope of this analysis. By synthesizing findings across disciplines and species, this review seeks to identify knowledge gaps and technological frontiers that can inform both veterinary practice and human medicine. This includes a critical evaluation of both the technical foundations and outcome data reported for each method.

## 2 Biological Basis for Sperm Sexing

Primary sex determination is the process by which male or female gonads are established. In mammals, this occurs owing to the presence of chromosomes, not the influence of the environment. Typically, individuals with two X chromosomes (XX) develop as females, while those with one X and one Y chromosome (XY) develop as males [21]. Gene mapping and chromosome painting have shown that the gene content and overall synteny of the X chromosome are highly conserved across eutherian (placental) mammals, and largely across therian mammals; this pattern, first articulated by Ohno, is known as Ohno's Law [22, 23]. By contrast, the Y chromosome has undergone substantial evolutionary change, including the accumulation of male‐specific and sex‐determining genes and the loss of ∼95% of its ancestral gene complement relative to the proto‐sex chromosome shared with the X in the therian ancestor (inferred from the conserved X and outgroup genomes, e.g., chicken), which also explains why it is smaller than the X chromosome [24, 25].

The Y chromosome contains the SRY genes, which induces the development of a male embryo. Several types of SRY genes have been identified, but their function remains the same regardless of species. It stabilizes the SOX9 protein in genetic males to initiate Sertoli cell differentiation and testicular cord formation [26]. In the absence of a Y chromosome (including SRY genes), the reproductive system develops as the ovaries. Some studies show that female sex‐determining genes—Rspo1(R‐spondin 1), Wnt4 (wingless‐type MMTV integration site family, member 4), and Foxl2 (forkhead box L2)—are crucial for the initiation of ovarian formation in females [27].

Y‐bearing and X‐bearing spermatozoa differ not only in the presence of sex‐determining chromosomes, but also in other factors potentially relevant to selecting spermatozoa with the desired sex chromosome. These include sperm mass [15], speed of movement [28], immunological characteristics, metabolic activity, surface protein expression, sensitivity to pH changes [29], DNA repair mechanisms, and heat sensitivity [30] (Table 2).

**TABLE 1:** Comparison of X and Y chromosomes in mammals.

Feature	X chromosome	Y chromosome
**Role in sex determination**	Typically leads to female development [26].	Contains the SRY gene, which initiates male development [26].
**Evolutionary Changes**	Highly conserved across species [22].	Underwent significant gene loss (∼95% of ancestral genes) and acquired male‐specific genes [24].
**Size and DNA content**	Larger; contains more DNA [25].	Smaller due to gene loss during evolution [24].
**Associated genes**	Includes genes critical for ovarian development [27].	Houses SRY and other male‐specific genes essential for testis and male reproductive structure development [26].
**Motility and swimming pattern**	Slower move; altered angular velocity in flow streams [38].	Faster move; may reach targets more quickly under certain conditions [38].
**Immunological characteristics**	Different surface proteins (e.g., 14 identified proteins differing between X and Y sperm) [41].	Contains unique proteins, such as H‐Y antigen, though its utility in sperm sexing is debated [44].
**Sensitivity to pH**	Enriched in acidic media, but results are inconclusive [29].	Contradictory findings exist; more research is needed to confirm pH influence on Y sperm enrichment [29, 46].
**Sensitivity to heat stress**	More resistant to heat stress [30]	More sensitive to heat stress [30].
**Application in sperm sexing**	Targeted in techniques utilizing weight, DNA content, and immunological markers for differentiation [18, 119].	Targeted in methods like MACS with scFv antibodies for specific sorting [42, 65].

**TABLE 2:** Comparative evaluation of sperm sexing methods.

Method	Efficiency / accuracy	Effects on sperm quality	Species‐specific applicability	Limitations	Maturity
Flow cytometry	High purity in cattle, lower in pigs; moderate in horses; limited data in humans; very limited in dogs [18, 52].	Potential impacts on motility/viability/DNA [53]	Strong: cattle; moderate: horses; limited: humans; challenging: pigs; very limited: dogs [18, 54, 55].	High cost; single‐cell sorting limits throughput [50]	Established (cattle); developing (horses, humans); limited (pigs, dogs) [49].
Immunomagnetic (MACS; scFv/SSAb)	Reported enrichment in cattle; variable across studies [68]	Generally preserves conventional quality metrics; requires validation of functional outcomes [42].	Demonstrated mainly in cattle [42]	Dependence on clinical‐grade, reproducible antibodies; target validation; bead removal/standardization; limited multi‐lab replication [70].	Promising [42]
Density gradient centrifugation	Inconsistent and generally low sex‐specific enrichment [76]	Possible membrane/mechanical damage; reductions in motility/viability and concentration [75].	Used historically in several species; ineffective in dogs [73].	Low cost and accessible but weak selectivity [74, 75].	Legacy / limited [75].
Swim‐up	Limited enrichment; occasional male‐biased fractions reported; inconsistent [83].	Gentle preparation; minimal adverse effects; low selectivity limits use for sexing [81].	Described in humans and cattle; limited coverage in other species [82, 83].	Simple and inexpensive, but low resolving power; not reliable for sexing [80].	Limited [80]
TLR7/8 ligand‐assisted	Effective separation reported in mice, cattle, and rams; ineffective in dogs [84, 85, 87]	Deliberate ATP/motility reduction in X‐bearing sperm; disadvantages when X‐enrichment is desired; possible spillover effects on Y with suboptimal dosing [90].	Strong species dependence: mice/cattle/rams yes; dogs no [85, 86, 87, 88, 89].	Operationally simple/cost‐effective; requires precise dosing/standardization; limited field validation [84].	Promising but species‐dependent [89].
Genome editing	Not a sorting method; proof‐of‐concept for redirecting gonadal fate in models (e.g., SRY knockout) [116, 117, 118].	Not applicable to semen processing; germline/embryo‐level intervention with major ELSI considerations [116, 117, 118].	Demonstrated in model species [116, 117, 118]	Ethical, legal, welfare, and regulatory barriers [116, 117, 118]	Speculative for routine sex control [116, 117, 118].

X‐ and Y‐chromosome‐bearing spermatozoa can be differentiated by their DNA content. In humans, X‐bearing spermatozoa have about 3% more DNA than Y‐bearing ones [31]; in cattle this difference is 3.8% [32], and in chinchillas, 7.5% [33].

Another difference between X‐ and Y‐bearing spermatozoa is their motility and swimming pattern [28]. Human spermatozoa can move at speeds of up to 1000–3000 µm/min [34]. While findings from other studies are inconclusive, many researchers believe that the difference in DNA content between X‐ and Y‐bearing spermatozoa may influence their motility and swimming pattern [35, 36]. Using an albumin gradient, Ericsson et al. [37] reported that Y‐bearing human spermatozoa reached the gradient's bottom before X‐bearing spermatozoa. However, these findings have been contested in subsequent studies, and the reliability of this method remains debated. For instance, a study by Sarkar et al. [38] observed that X‐bearing spermatozoa moved more slowly than Y‐bearing ones in flow streams, suggesting potential differences in motility, but not conclusively validating the Ericsson approach.

Immunological sperm sexing techniques are based on the distinct proteins found on the surface of X‐ and Y‐bearing spermatozoa [17]. If these proteins can be isolated, antibodies against such markers could potentially be used to identify X‐ and Y‐chromosome bearing cells [39]. A male‐specific protein, the H‐Y antigen has attracted attention as a possible marker for distinguishing between Y‐ and X‐bearing spermatozoa, but results have been contradictory [40]. Consequently, this protein is not currently used for sperm sexing. Chen et al., have identified 14 proteins that are differentially expressed between X‐ and Y‐bearing spermatozoa, which may contribute to their distinct phenotypes [41]. These proteins are involved in various biological processes, including energy metabolism, stress resistance, cytoskeletal composition, serine protease activity, sperm–oocyte binding and fusion, and zygote development. These differences help explain the unique functional characteristics of each sperm type during fertilization and early embryonic development [41].

Another immunological method was evaluated by Sringarm et al. [42], who examined the effectiveness of magnetic‐activated cell sorting (MACS) using Single‐Chain Fragment Variable (scFv) antibodies attached to magnetic microbeads targeting potential Y‐chromosome‐bearing sperm surface antigens, for separating bovine spermatozoa. The method's impact on kinematic properties, sperm quality, and the ratio of X‐ to Y‐bearing spermatozoa was assessed. The technique showed excellent efficiency in differentiating between X‐ and Y‐spermatozoa, while preserving sperm quality [43]. Different studies have identified sperm‐sex‐specific antibodies (SSAb) in rabbit antisera directed against bovine sex‐sorted sperm. These antibodies have been used to isolate sex‐specific proteins associated with either X‐ or Y‐bearing bovine spermatozoa. Findings suggested that a 30‐kDa protein may be uniquely associated with bovine X‐bearing spermatozoa, indicating its potential as a marker for immunological sexing methods [44]. However, relative to established DNA‐content flow cytometry in bulls‐which exploits the ∼3%–4% higher DNA content of X‐bearing sperm and routinely yields high‐purity X or Y fractions‐the MACS/scFv and SSAb approaches remain exploratory: reported enrichments are variable, have not yet matched FACS‐level purity or field fertility, and lack robust multi‐laboratory replication [42, 44, 45].

In the testis, mammalian spermatozoa are immotile; as they move through the epididymis, they become motile in response to environmental stimuli. Among these, ionic concentrations—particularly pH levels—are essential for regulating sperm maturation and the development of the motility required for fertilization [28]. X‐ and Y‐bearing spermatozoa may differ in their pH sensitivity, but results are conflicting. Oyeyipo et al. found successful enrichment of X‐bearing spermatozoa after incubation in acidic media [29]. On the other hand, Raval et al. did not find the influence of pH on the percentage of X‐ or Y‐bearing spermatozoa [46].

It has been suggested that different types of male germ cells vary in their resistance to brief, mild heat treatment. Pérez‐Crespo et al. [30] applied thermal stress by exposing male mice to elevated scrotal temperatures (42°C for 30 min daily over several days). This heat shock affected epididymal spermatozoa and led to a distortion of the offspring sex ratio, likely by differentially impairing the viability of Y‐bearing spermatozoa. The altered sex ratio was confirmed by evaluating the proportion of male and female embryos after mating [30]. These finding imply that X‐ and Y‐bearing spermatozoa respond differently to heat stress: Y‐bearing spermatozoa exhibit reduced viability and are more susceptible to apoptosis, while X‐bearing spermatozoa demonstrate greater resilience and longer survival under elevated temperature conditions [30, 47]. Other studies suggest that Y‐bearing spermatozoa are more susceptible than X‐bearing ones to physiological changes occurring during liquid storage, such as cold shock and oxidative stress [47]. For example, You et al. reported that ejaculated Y‐bearing spermatozoa stored at 4°C showed a higher rate of apoptotic markers compared to X‐bearing ones, as measured by Annexin V staining [47]. This increased susceptibility to apoptosis in vitro may explain their faster decline in fertilization potential during semen preservation [47].

## 3 Methods of Sperm Sexing

### 3.1 Flow Cytometry

Flow cytometry is the most common, reliable, precise and cost‐effective method for sperm sexing [48]. It separates X‐ and Y‐bearing spermatozoa based on differences in DNA content using fluorescent dyes. This method has become a standard in agricultural breeding programs aimed at controlling offspring sex to optimize production [49].

**FIGURE 1:**
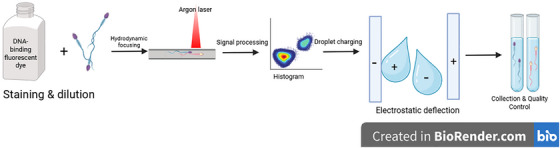
**Flow cytometric sex sorting of sperm based on DNA content. Created in** https://BioRender.com.

The separation process involves multiple stages (Figure 1). It begins with sample preparation, during which spermatozoa are stained with DNA‐binding fluorescent dyes that recognize nucleic acids and enable discrimination based on DNA content [50]. The stained cells travel through a fluidic system, suspended in a saline solution—often supplemented with 1% BSA and EDTA [50]. A piezoelectric crystal separates the cells into distinct droplets, so that each contains a single spermatozoon. After entering the optical system, the droplets are exposed to an argon laser, which excites the fluorescent dyes [51]. One detector measures the amount of DNA, while another the cell's orientation. Two photomultipliers are used to detect the fluorescence. After processing the fluorescence signals, the electronic system creates a histogram that shows two subpopulations according to fluorescence intensity: X‐bearing sperm are on the right side of the histogram, indicating higher fluorescence, and Y‐bearing sperm are on the left, indicating lower fluorescence [19]. This fluorescence‐based differentiation enables electrostatic separation, where droplets are charged differently based on sperm type; X‐bearing sperm are positively charged and directed toward a negative plate, while Y‐bearing sperm are negatively charged and deflected toward a positive plate [50].

This method has successfully produced offspring in various species, including sheep, cattle, rabbits, pigs, horses, dogs, cats, and dolphins [18]. However, commercially available sexed semen exists only for cattle, largely due to demand for female calves in dairy farming, where females are valued for their milk production. This allows dairy farmers to enhance productivity by selectively increasing the female population in their herds, making bovine sexed semen the primary commercial product of sperm sexing technologies [52].

Although sperm sexing by flow cytometry was initially demonstrated to be feasible for pigs, it is still remains impractical for other livestock species. This is mostly because pigs have a unique uterine anatomy that necessitates a high sperm count for optimal fertility. Moreover, the flow cytometry sorting rate is insufficient to produce the necessary volume of the sperm suspension per session. As a result, sexed boar sperm is not commercially viable in the swine artificial insemination industry [53].

In stallions, sperm sorting efficiency varies significantly between individuals, often due to the proportion of viable spermatozoa in each sample. Despite technological improvements that have increased throughput to 70–80 million cells per hour for certain species [54], the single‐cell nature of flow cytometry continues to limit scalability. In practice, low‐dose, deep‐horn insemination is commonly used in horses, with pregnancy rates ranging from 10%–40% when 5–25 million sex‐sorted sperm are applied. In equine reproduction, hysteroscopic insemination of concentrated doses (e.g., 1 million spermatozoa in 100–250 µL) helps maintain acceptable fertility, as high dilution may negatively affect success rates [54].

To date, only one study has reported flow cytometric sorting on canine sperm, conducted on just three dogs [55]. This indicates a significant need for further research in this area.

In human reproduction, there are few reports on sperm sexing by flow cytometry [15, 56, 57]. Satisfactory results have been obtained in all studies, with authors concluding that semen processed by this method could be effectively used in both in vitro fertilization and intrauterine insemination [56, 57, 58].

### 3.2 Magnetic‐Activated Cell Sorting (MACS)

Magnetic‐activated cell sorting (MACS) is widely used in andrology and across many other biomedical fields. Its applications include as follows: selection of stem cells [59], research in the field of immunology [60] and oncology [61], and in prenatal diagnosis [62], microbial studies [63] and widely in andrology [64, 65, 66].

**FIGURE 2:**
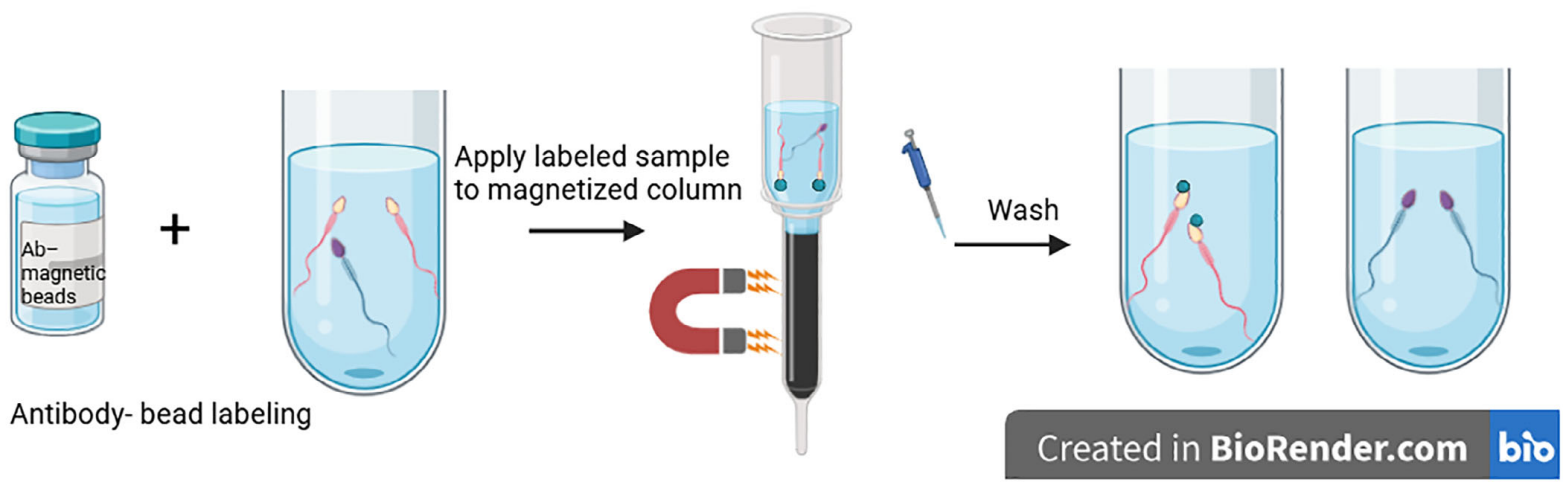
**Magnetic‐activated cell sorting (MACS) for sperm sexing. Created in** https://BioRender.com.

The MACS procedure begins with sample preparation, which includes washing and dilution (Figure 2). During this step, spermatozoa are stained with specific antibodies conjugated to magnetic particles, which bind to surface proteins differentially expressed on X‐ or Y‐chromosome‐bearing spermatozoa, such as the H‐Y antigen [42]. These antibodies, attached to the magnetic beads, create a population of magnetically labeled spermatozoa that can be selectively separated as they pass through a magnetic column or separator [67].

Currently, the application of MACS is primarily experimental and not yet widely used in commercial breeding. To date, it has been applied only to bovine semen. Sringarm et al. used scFv antibody against Y‐chromosome‐bearing spermatozoa (Y‐scFv) conjugated to magnetic microbeads [42]. This approach yielded sperm samples with up to 82.7% X‐bearing cells and up to 81.4% in the Y‐bearing. Moreover, this method preserved motility, kinematic parameters, and overall quality of X‐bearing spermatozoa, although Y‐bearing spermatozoa showed reduced quality after sorting [42]. A second study using the same antibodies reported comparable results [68]. In that study, motility and quality of X‐enriched sperm were similar to those of conventional (CONV) semen samples whereas the Y‐enriched fraction exhibited significantly lower sperm quality than both X‐enriched and CONV samples. Sperm distribution analysis revealed that X‐bearing sperm made up to 79.5% of the X‐enriched fraction, while Y‐bearing sperm accounted for up to 78.6% of the Y‐enriched fraction [68]. The results from both research teams indicate that MACS is a reliable and repeatable method for sperm sexing. However, because MACS has only been tested on bovine semen, additional research is necessary across other species, including humans. Regarding feasibility for large‐scale applications, several practical considerations remain. First, throughput and purity need to be demonstrated at production‐relevant scales: MACS can process large cell numbers in bulk, but sex‐enrichment depends on antigen specificity and may not reach the purities routinely achieved by DNA‐content flow cytometry [69]. Second, scalability hinges on reagent supply and standardization of clinical‐grade, reproducible antibodies (or scFv) targeting validated, surface‐exposed epitopes, as well as robust bead/column workflows with minimal batch‐to‐batch variability [70]. Third, process integration and costs (automation, single‐use closed systems, bead carryover testing, and rapid bead removal) must be addressed to enable high‐throughput semen processing with acceptable turnaround times and cost per insemination dose. Fourth, biological safety and performance require evidence that labeling and magnetic separation do not impair sperm function, fertilization competence, or offspring health, and that results are reproducible across bulls, lots, and laboratories [71]. Finally, for human use, regulatory compliance (GMP‐grade reagents, validated cleaning/removal steps, and clinical trials) and ethical oversight would be necessary [72]. Collectively, these factors suggest that MACS‐based sperm sexing is promising but still requires rigorous validation before it can be considered ready for large‐scale deployment.

### 3.3 Centrifugation

The Y chromosome is significantly smaller than the X chromosome [15]. The Percoll density gradient centrifugation method is a commonly used technique for separating X‐ and Y‐bearing spermatozoa by exploiting differences in cell mass and head size. However, this method can negatively affect sperm quality, as the sexing process may lead to membrane damage, which in turn compromises both motility and viability. Recent studies suggest that this technique not only reduces motility and viability but also lowers the overall concentration of spermatozoa [73].

The Percoll method is most widely used in bovine andrology and reproductive biotechnology [74, 75]. Although it is relatively cost‐effective, the centrifugation protocols have been shown to impair multiple sperm quality parameters during the sexing process. Despite these limitations, the method is still considered a viable approach for sperm sexing in certain contexts [74].

This technique has also been tested for canine sperm sexing; however, the Percoll centrifugation method did not lead to significant enrichment of either X‐ or Y‐bearing spermatozoa [76]. Several studies have also investigated the use of this method in human sperm sexing. Iizuka et al. reported a 94% purification of X‐bearing sperm [77], while Kaneko et al. observed lower efficiency—about 73.1% [78]. Although those findings provided valuable early insights, advancements in methodology highlight the need for updated research in this area. To date, only one recent study has evaluated the use of the Percoll gradient for sexing human semen, concluding that the method is not ideal [79]. The limited number of current studies may reflect the potential impact on sperm quality and sperm–egg interaction, fertilization, and embryo development [75].

### 3.4 Swim‐Up Techniques

Swim‐up is a simple method of sperm preparation in which motile sperm, more than 90%, are isolated, and at the same time, dead sperm and cryoprotectant agents are removed [80]. Some work has been reported on using swim‐up as a method for isolating X and Y sperm, exploiting their differential motility to achieve separation [81, 82]. This method has been tried on semen of bulls [82] and humans [83].

In both species this method was effective, resulting in 81% male offspring in humans [83]. In bovine samples, the motile sperm recovery rate for the presumptive X chromosome‐bearing sperm population in the lower supernatant fraction was 5.2% [81]. In the current literature, there is no data on the use of this method of sperm sexing in dogs, pigs, or horses.

### 3.5 TLR7/8 Ligand‐Assisted Sperm Sexing

**FIGURE 3:**
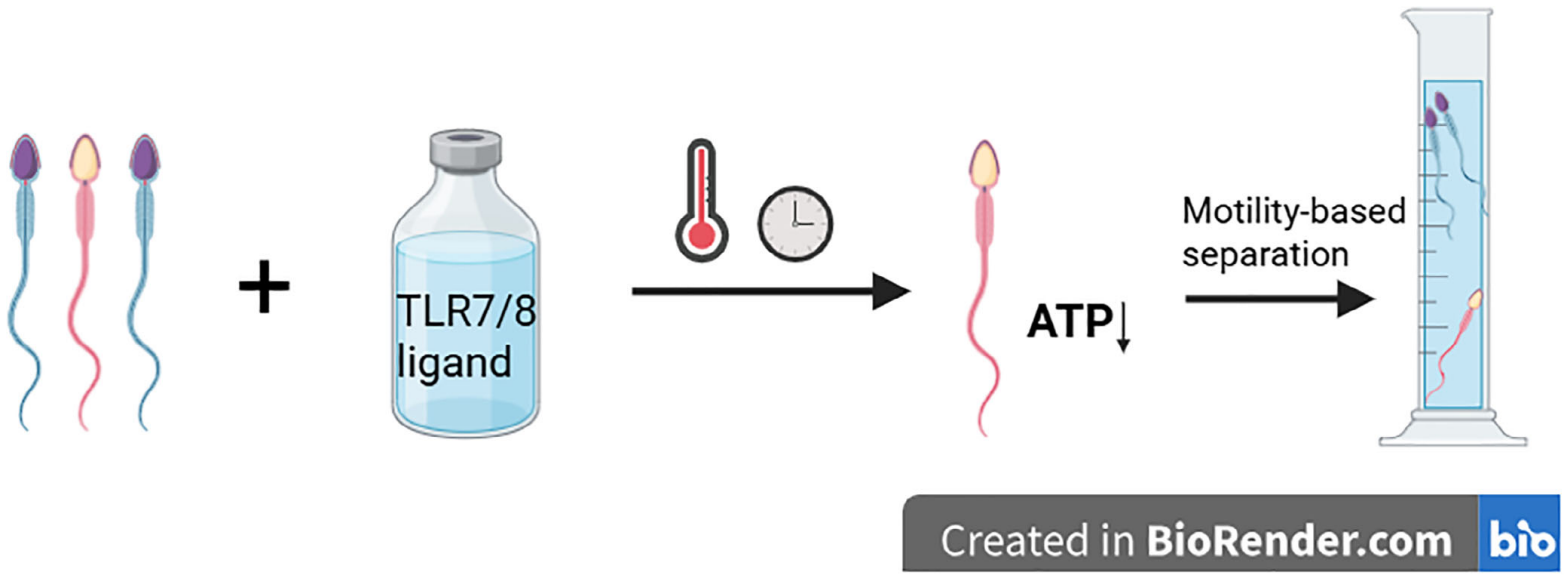
**TLR7/8 ligand‐assisted sperm sexing. Created in** https://BioRender.com.

In recent years, researchers have increasingly focused on the discovery that Toll‐like receptors 7 and 8 (TLR7/8), which are membrane‐bound receptor proteins, expressed exclusively on X‐bearing spermatozoa and are absent on Y‐bearing ones. This finding has led to the development of a novel sperm sexing method involving the use of TLR7/8 ligands (Figure 3). These ligands have been shown to selectively reduce the motility of X‐bearing sperm by decreasing their ATP levels, thereby enabling their separation from Y‐bearing sperm based on differential movement [84].

Setiawan et al. investigated the effect of a TLR7/8 ligand on the motility of putative X‐ and Y‐chromosome‐bearing sperm in rams. Their study confirmed that activation of TLR7/8 receptors allows effective separation via swim‐up technique [84]. Similar studies have been performed on mice [85, 86], cattle [87, 88], and dogs [89]. This method appears to be particularly promising in species such as cattle, rams, and mice, where high success rates of sperm sexing have been reported. In all cases, authors observed effective separation of X‐ and Y‐bearing spermatozoa [84, 85, 86, 87, 88]. However, in dogs, the method has proven to be ineffective [89].

This method offers several notable advantages. It is highly specific, cost‐effective, and does not require advanced or specialized laboratory equipment. However, it also presents certain limitations. Most importantly, its effectiveness appears to be species‐dependent‐to‐date; it has not yielded successful results in dogs [89]. This X‐specific motility suppression aids Y‐selection but is disadvantageous when X‐enrichment is the goal; additionally, suboptimal dosing/exposure may impair Y‐bearing sperm function [90].

## 4 Molecular Techniques—Results Evaluation

The results of sperm sexing can be evaluated in vivo [83] or using molecular techniques [55, 82]. To date, the most commonly used method for evaluating the results of semen sexing is PCR testing, which can only be used for research or for methodology validation, as it is not possible to use the tested cells for fertilization. The method is based on detection of X chromosome‐specific (PLP) and Y chromosome‐specific (SRY) regions [91]. Two PCR methods have been evaluated so far: simplex [92] and multiplex real time PCR [91]. The multiplex assay is as effective as the simplex assay in determining sperm sex ratio in both reference plasmids of known ratio and actual semen samples [91]. However, Cray et al. suggested that quantitative PCR is insensitive in this context because Ct‐based, relative quantification has limited resolution for detecting small differences in X:Y abundance (e.g., distinguishing ∼95:5 from ∼90:10), particularly in sex‐sorted, frozen–thawed samples and due to dependence on amplification efficiency and standard curves [93]. They optimized and validated a multiplexed digital droplet PCR (ddPCR) assay that employed a copy counting method to quantify sex skew (the ratio of X to Y chromosomes) in frozen‐thawed bovine sexed semen. This assay analyzes at least 1000 cells per sample quantifying X and Y chromosome copy numbers alongside GAPDH, an autosomal gene used as an internal control to verify the total cell count [93].

Fluorescence in situ hybridization (FISH) is a single‐cell assay that is accurate and cost‐effective. It can determine the sex of individual spermatozoa and quantify aneuploidy in semen samples. For sexing, at least one sex chromosome–specific probe is required, ideally with distinct probes for X and Y. 94 FISH can also assess broader chromosomal abnormalities in spermatozoa [94, 95].

The latest technology with broad applications in science and medicine is Next Generation Sequencing (NGS) [96], which has not been used for sperm sexing evaluation but potentially it could be useful. NGS offers a more comprehensive assessment of genetic material than FISH [97]. In addition, compared with PCR/ddPCR, NGS can multiplex many sex‐linked and quality‐control markers at once, quantify aneuploidy or structural variants, estimate minor carryover of the non‐target fraction from read counts, and detect off‐target effects introduced by processing [96]. However, for single‐marker sex ratio assessment, PCR/ddPCR remains simpler and usually more cost‐effective per sample. NGS becomes competitive mainly when libraries are highly multiplexed (e.g., tens to hundreds of samples per run) and when broader genomic information is required beyond presence/absence of SRY or a small panel of targets [98].

Potentially, specific markers in sperm could be assessed using NGS. Evaluation of specific markers so far has been carried out on *Spinibarbus hollandi* and the accuracy was 100% [99]. NGS is suitable for sex‐linked gene (SRY) detection as confirmed by Dong et al. among others [100]. NGS can be performed on various types of samples, even for semen [101], so potentially this method in the future could be used for evaluation the chromosomes after sexing.

## 5 Applications Across Species

Sperm sexing is used in several countries such as the United States, Canada, Australia, and a number of European countries [102]. The dairy industry is focused on producing female calves (from X‐bearing spermatozoa) [52]; however beef industry concentrates on producing males (from Y‐bearing spermatozoa) [103].

Sex preselection of offspring would be extremely beneficial for reproductive management in pig production, allowing for planned mattings according to male or female breeding lines. However, its application in swine—as in other livestock species—remains limited due to economic and technical challenges [104].

Currently, equine semen sexing is not commercially available, although it is potentially the future of equine reproduction [105]. For instance, it may be economically beneficial to obtain successful thoroughbred male foal for racing than to replicate the genetic traits of the dam that produced him. Both sexes are valuable, but mares can produce only one foal per year, while a single stallion can sire multiple offspring within the same period [106].

Semen sexing in dogs is also not commercially available. In this species, there is no consistent preference among breeders regarding the sex of the offspring. That said, there are some functional sex‐based preferences: for example, males may be favored in sheep herding [93], and male hunting dogs are often preferred due to their generally bolder temperament and higher activity levels compared to females [107]. On the other hand, many pet buyers prefer females, likely due to perceived differences in behavior and manageability. This lack of a strong commercial driver may explain the limited focus on developing sperm sexing methods for dogs, and only a few research studies have been published to date [55, 76].

In human medicine, semen sexing raises ethical questions. Choosing a child's sex before birth is controversial, as it raises ethical concerns similar to those highlighted in human rights discussions regarding racial preference, which is strongly prohibited. Sex selection, favoring one sex over the other, can be seen as discriminatory, potentially diminishing the value of the opposite sex within society. Sex selection and sex preference are often viewed as forms of discrimination and prejudice, with one of their unintended consequences being sex imbalance [72].

On the other hand, a number of sex‐linked diseases have been recognized and described in humans [108, 109]. Some diseases are caused by mutations in single genes, such as hemophilia [110] and Duchenne muscular dystrophy [111], which are X‐linked (sex‐linked) disorders. Sperm sexing allows the selection of sperm that do not carry parents' defects in the vulnerable sex chromosomes thus reducing the probability of passing on these conditions to the next generations. This approach allows for a proactive and precise preconception intervention by selecting X‐ or Y‐bearing sperm before fertilization. This solution offers a targeted solution that could greatly alleviate the burden of sex‐linked genetic disorders on affected families and populations.

The regulation of sperm sexing in humans varies widely across countries, reflecting diverse cultural and ethical perspectives. In the United States, non‐medical sperm sexing for purposes like family balancing is allowed and commercially available [50]. In contrast, many European countries, including the United Kingdom and Germany, restrict sperm sexing to medical uses, such as preventing sex‐linked genetic disorders, as outlined in conventions like the Council of Europe's Article 14 on Biomedicine [112]. By comparison, India imposes a blanket prohibition on sex determination and sex selection before and after conception, without a general medical exception, which is stricter than both the United States and the typical European “medical‐only” model. China also bans non‐medical fetal sex determination and sex‐selective abortion; enforcement focuses on curbing non‐medical use, with policies periodically reaffirmed nationwide [50]. These disparities highlight how local laws and values influence the ethical landscape of assisted reproductive technologies.

## 6 Future Directions and Emerging Technologies

Due to the fact that sperm sexing does not yield satisfactory results in many species, and the demand for sex‐specific offspring continues to grow, new technologies are emerging (Table 3). Importantly, the magnitude and nature of these effects are technique‐dependent, as outlined above for the different approaches to sperm sexing. Sperm sexing can negatively impact sperm quality, which may contribute to reduced pregnancy rates. Techniques such as flow cytometry have the potential to cause DNA damage, reduce motility, and impair sperm viability [113]. Other methods, including density gradient centrifugation, may induce mechanical damage to sperm membranes, further compromising their fertilization potential [114]. Studies have demonstrated that these effects can lower the overall quality of sex‐sorted spermatozoa, which correlates with diminished fertility outcomes, including reduced conception rates and fewer live births [115]. On the other hand, MACS can be an example of a method with little or no effects on fertility [67].

**TABLE 3:** Comparison of sperm sexing methods across species.

Species	Methods	Efficiency	Challenges	Applications
**Human**	Flow cytometry, Percoll density gradient centrifugation, Swim‐up [58, 77]	High (90%–95%) [58]	Ethical concerns, high cost, potential DNA damage [72]	Sex selection in assisted reproduction [58]
**Horse**	Flow cytometry [18]	Moderate (10%–40%) [105]	Limited sperm availability, reduced sperm motility post‐sorting [105]	Breeding programs, conservation of rare breeds [106]
**Swine**	Flow cytometry [113]	Low [113]	High cost, low sorting speed, sensitivity to handling stress [113]	Optimizing litter composition for breeding [104]
**Cattle**	Flow cytometry (most common), magnetic‐activated cell sorting (MACS), swim‐up [17, 18, 102]	High (∼90%) [1]	Equipment cost, reduced post‐sort fertility due to stress [1, 102]	Dairy and meat production optimization [12, 52]
**Dogs**	Flow cytometry (experimental), Percoll density gradient centrifugation [55, 76]	Low to moderate [55, 76]	Low research focus [55, 76]	Conservation breeding, potential in companion animal reproduction [55, 76]

Emerging genome‐editing technologies like Zinc Finger Nucleases (ZFN) [116], Transcription Activator‐Like Effector Nucleases (TALENs) [117], and the CRISPR/Cas system [118] offer significant potential for influencing sex determination at the genomic level by directly editing DNA in key sex‐determining loci or their cis‐regulatory elements (e.g., inducing loss‐ or gain‐of‐function) [117]. The sex‐determining region on the Y chromosome (SRY) is the primary genetic switch for male development. Studies in mice and rabbits have shown that knocking out the SRY gene suppresses testis formation in fetal gonadal tissue, leading to a female phenotype [117]. These advancements present promising opportunities to pre‐determine sex; however, further research is required to fully harness their practical applications.

## 7 Conclusion

Sperm sexing is well‐established in cattle and has markedly improved breeding efficiency, whereas performance in pigs and horses remains below bovine benchmarks and controlled evidence in dogs is still limited. Current methods face trade‐offs‐flow cytometry offers the most reliable enrichment but is costly and can affect sperm quality; density gradients provide inconsistent gains; immunologic and ligand‐assisted approaches are promising yet not fully validated and often species‐dependent. Near‐term progress is most likely from engineering refinements to flow sorting and from immunomagnetic/ligand strategies contingent on robust, sex‐specific surface targets; genome‐editing concepts remain speculative for routine use due to ethical and regulatory constraints. Responsible deployment should be evidence‐based and regulated, prioritizing clear benefits for animal welfare and conservation (not merely commercial gains) while avoiding sex‐ratio distortions and social harms.

## Author Contributions

KD was responsible for writing the original draft of the manuscript. PJ reviewed and edited the manuscript, providing critical revisions and improvements, and served as the supervising author for the project. Both authors approved the final version of the manuscript for publication.

## Funding

The publication was (co)financed by Science development fund of the Warsaw University of Life Sciences—SGGW.

## Conflicts of Interest

The authors declare no conflicts of interest.
